# Biodistribution and Subcellular Localization of an Unnatural Boron-Containing Amino Acid (*Cis*-ABCPC) by Imaging Secondary Ion Mass Spectrometry for Neutron Capture Therapy of Melanomas and Gliomas

**DOI:** 10.1371/journal.pone.0075377

**Published:** 2013-09-18

**Authors:** Subhash Chandra, Rolf F. Barth, Syed A. Haider, Weilian Yang, Tianyao Huo, Aarif L. Shaikh, George W. Kabalka

**Affiliations:** 1 Department of Biomedical Engineering, Cornell University, Ithaca, New York, United States of America; 2 Department of Pathology, The Ohio State University, Columbus, Ohio, United States of America; 3 Departments of Radiology and Chemistry, The University of Tennessee, Knoxville, Tennessee, United States of America; University Hospital Hamburg-Eppendorf, Germany

## Abstract

The development of new boron-delivery agents is a high priority for improving the effectiveness of boron neutron capture therapy. In the present study, 1-amino-3-borono-cyclopentanecarboxylic acid *(cis*-ABCPC) as a mixture of its L- and D- enantiomers was evaluated *in vivo* using the B16 melanoma model for the human tumor and the F98 rat glioma as a model for human gliomas. A secondary ion mass spectrometry (SIMS) based imaging instrument, CAMECA IMS 3F SIMS Ion Microscope, was used for quantitative imaging of boron at 500 nm spatial resolution. Both *in vivo* and *in vitro* studies in melanoma models demonstrated that boron was localized in the cytoplasm and nuclei with some cell-to-cell variability. Uptake of *cis*-ABCPC in B16 cells was time dependent with a 7.5:1 partitioning ratio of boron between cell nuclei and the nutrient medium after 4 hrs. incubation. Furthermore, *cis*-ABCPC delivered boron to cells in all phases of the cell cycle, including S-phase. *In vivo* SIMS studies using the F98 rat glioma model revealed an 8:1 boron partitioning ratio between the main tumor mass and normal brain tissue with a 5:1 ratio between infiltrating tumor cells and contiguous normal brain. Since *cis*-ABCPC is water soluble and can cross the blood-brain-barrier via the L-type amino acid transporters (LAT), it may accumulate preferentially in infiltrating tumor cells in normal brain due to up-regulation of LAT in high grade gliomas. Once trapped inside the tumor cell, *cis*-ABCPC cannot be metabolized and remains either in a free pool or bound to cell matrix components. The significant improvement in boron uptake by both the main tumor mass and infiltrating tumor cells compared to those reported in animal and clinical studies of *p*-boronophenylalanine strongly suggest that *cis*-ABCPC has the potential to become a novel new boron delivery agent for neutron capture therapy of gliomas and melanomas.

## Introduction

Boron Neutron Capture Therapy (BNCT) is a binary modality that has been used to treat a variety of malignancies, the most important of which have been high grade gliomas and recurrent cancer of the head and neck region [1]. The main requirements for BNCT are the selective targeting of tumor cells with sufficient quantities of ^10^B atoms (10^9^/cell or ∼20 µg/g) and neutron irradiation with either epithermal (E≅ 10,000 eV) or low-energy thermal neutrons (E_th_<0.4 eV) depending upon the depth of the tumor. BNCT is based on the neutron capture and fission reactions [^10^B(n, α)^7^Li] that occur when ^10^B atoms capture thermal neutrons and undergo instantaneous nuclear fission to produce high linear energy transfer (LET) alpha particles (stripped down ^4^He atoms) and recoiling ^7^Li nuclei. These particles have short path-lengths (5 µ for ^7^Li and 9 µ for ^4^He), which is approximately the diameter of a single cell. The average LET is high (^7^Li, 162 keV/µ; ^4^He, 196 keV/µ), and this results in densely ionizing radiation restricted to the path length of each particle [2], [3]. Cell killing is enhanced by localization of ^10^B in the nucleus, where high LET radiation has a greater probability of damaging the DNA [4], [5]. BNCT is potentially capable of killing individual cancer cells while sparing contiguous normal tissues. To minimize normal tissue injury, the quantity of boron in tumor cells should exceed that found in surrounding normal cells by at least a factor of three [6], [7]. Consequently, study of the distribution of ^10^B atoms at the subcellular scale is critical for the development of new boron delivery agents for neutron capture therapy (NCT) [1], [8].

Over the last several decades, there have been only two drugs used clinically for BNCT, L-*p*-boronophenylalanine (BPA) and disodium mercapto-*closo*-dodecaborate (BSH). Despite the fact that their selectivity to tumor cells is less than ideal, they have become useful “second” generation drugs for BNCT [1]. Clinical trials involving patients with high grade gliomas in the U.S., Japan, and Europe have demonstrated that BNCT is safe, requires only one or two irradiations, and has fewer side effects than conventional external beam photon irradiation. Furthermore, the median survival time of 12–18 months compares favorably to that of conventional radiotherapy in combination with temozolomide [1], [9]–[11]. Impressive clinical results also have been observed in patients with recurrent tumors of the head and neck region [1]. As clinical trials of BNCT continue worldwide using either BPA or BSH, either alone or in combination, it has been apparent for many years that new and more selective boron delivery agents are needed to improve the clinical efficacy of BNCT.

An ideal boron delivery agent should deliver sufficient quantities of ^10^B atoms selectively to all tumor cells, irrespective of their cell cycle status, and should be non-toxic to normal cells. Despite extensive efforts to develop new delivery agents to selectively target tumor cells [12], [13], based on their enhanced metabolism and upregulation of amino acid transporters, higher rates of proliferation, over expression of certain types of surface receptors, etc. [14]–[19], none have gone beyond preclinical studies. The boron containing unnatural cyclic amino acids (UNAAs) are a class of water soluble compounds that have shown the potential to be the new and more efficient boron carriers [20], [21]. A recent evaluation of several UNAAs, based on homogenized tissue boron measurements with inductively coupled plasma–optical emission spectroscopy (ICP-OES), identified *cis-*ABCPC to be far superior than BPA in producing tumor-to-blood boron ratios in B16 mouse melanoma model and tumor-to-normal brain boron ratios in F98 rat glioma model [22]. These observations provide compelling support for subcellular scale characterization of *cis*-ABCPC, so that the boron-targeting of the nucleus of individual tumor cells and boron-partitioning between the infiltrating tumor cells and the normal brain tissue can be assessed. These are essential measurements if this compound is to become a new candidate drug in BNCT.

Secondary ion mass spectrometry (SIMS) techniques have become valuable tools in biology and medicine for the localization of elements, molecules, and isotopically labeled compounds and therapeutic drugs in cells and tissues [23]–[28]. In the present study, a dynamic SIMS based technique of ion microscopy was employed for the detection of boron atoms from *cis*-ABCPC at a subcellular scale resolution in melanoma and high grade glioma models. The CAMECA IMS-3f SIMS instrument used in this study was capable of producing visual images of gradients of any element from H to U with ppm-to-ppb sensitivity and image lateral resolution of 500 nm [23]. The technique has been standardized for quantitative subcellular scale analysis of boron isotopes in both *in vitro* and *in vivo* models for BNCT studies [23], [29]–[31]. The B16 mouse melanoma and F98 rat glioma models were used for the evaluation of *cis*-ABCPC as a new boron delivery agent for BNCT. Our *in vitro* and *in vivo* data suggest that further studies of this compound are warranted to assess its potential.

## Materials and Methods

### Materials

**Figure 1:**
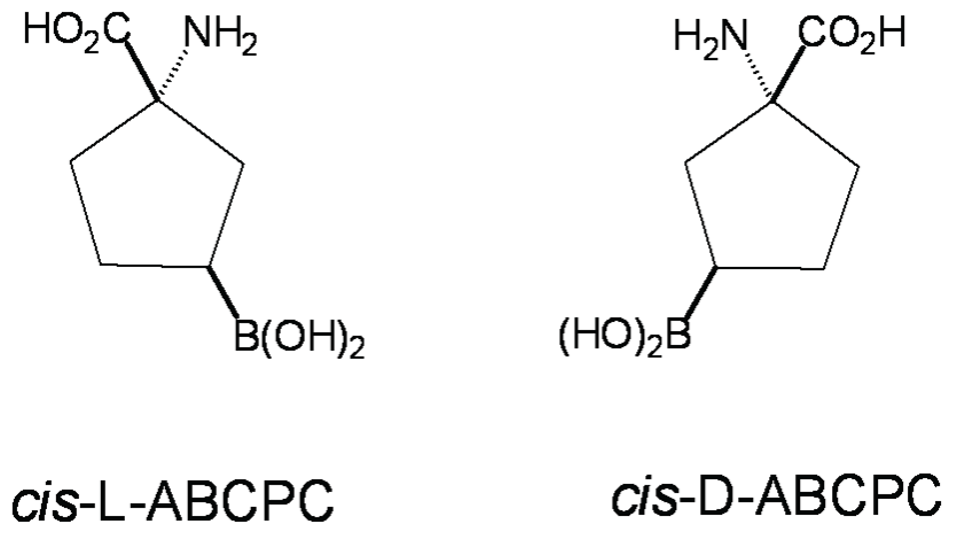
Chemical Structures of L- and D- enantiomers of *cis*-1-amino-3-borono-cyclopentanecarboxylic acid (*cis*-ABCPC).

The *cis*-ABCPC, as a mixture of L- and D- enantiomers (Fig 1), was synthesized containing natural isotopic abundance of boron (80 atom% ^11^B, 20 atom% ^10^B) [21]. The *cis*-ABCPC was further purified by removing the contaminant of ammonium chloride produced during its synthesis [22]. The chemical structures of L- and D- enantiomers of *cis*-ABCPC are shown in Fig. 1. Since *cis*-ABCPC is water soluble, it was dissolved directly in Dulbecco’s Modified Eagle Medium (DMEM) or phosphate buffer solution for various experiments discussed in this section. Hematoxylin and eosin (H&E) stain and 5-bromo-2′-deoxyuridine (BrdU) were purchased from Sigma-Aldrich, Inc. (St. Louis, MO). Latex beads (11 µm in diameter) were purchased from Duke Scientific (Palo Alto, CA). Polished high-purity N-type semiconductor-grade silicon wafers were purchased from Silicon Quest International (Santa Clara, CA). The 6-well Cell Culture Plates were purchased from Corning (Corning, NY).

### SIMS boron distribution studies of *cis*-ABCPC in B16 mouse model for human melanoma

All of the animal studies were carried out in strict accordance with the recommendations in the *Guide for the Care and Use of Laboratory Animals* of the National Institutes of Health and our protocol was approved by the Institutional Animal Care and Use Committee of The Ohio State University (IACUC protocol # 2007A0261-R1). Female C57BL/6 mice were injected subcutaneously (s.c.) into the right flank with 10^6^ B16 melanoma cells. Biodistribution studies were initiated ∼10 days later, at which time the tumors had attained a diameter of ∼1 cm. *Cis*-ABCPC was dissolved in phosphate buffered saline (PBS) pH 7.4 for administration. A dose equivalent of 24 mg B/kg b.w. was administered via an intraperitoneal injection (i.p.) to groups of four mice. They were euthanized at 1, 2.5, and 4 hr. post injection and a portion of their tumors were frozen in the vapor phase of liquid nitrogen for SIMS analysis to determine subcellular localization of ^11^B from *cis*-ABCPC. Serial 4µ thick cryosections were used for correlative optical and SIMS imaging analysis of the microdistribution of boron. Sections for histological examination were mounted on glass slides and stained with H & E. Cryosections for SIMS analysis were attached to silicon wafers (∼1 cm^2^), freeze-dried, and sputter coated with a 10 Å layer of Au/Pd for enhancing their electrical conductivity for SIMS imaging analyses.

### SIMS studies of *cis*-ABCPC in B16 melanoma cells in culture: evaluation of the time dependence of boron uptake, boron retention, and the S-phase boron targeting

The B16 melanoma (ATCC CRL No. 6322) was purchased from the American Type Culture Collection, Manassas, VA 20108. B16 melanoma cells were maintained in DMEM supplemented with 10% fetal bovine serum, L-glutamine, and antibiotics. The cells were grown on the polished surface of N-type semiconductor grade silicon wafers with ∼1 cm^2^ surface area [32]. Approximately 2×10^5^ cells were seeded per well of 6-well cell culture plates containing 5 silicon wafers in each well. After the cells reached ∼70% confluency on the silicon wafers, they were divided into five groups. The cells were exposed to 30 ppm boron equivalent of *cis*-ABCPC (as a mixture of L- and D- enantiomers) for 1, 2.5, 4 hr., and a 4 hr. drug treatment, followed by a 30 min. exposure to DMEM alone in order to determine intracellular retention. The fifth group consisted of cells exposed to 30 ppm boron equivalent *cis*-ABCPC in the presence of 50 µM bromodeoxyuridine (BrdU) for the detection of DNA-synthesizing S-phase cells among the asynchronous population. After the respective treatments the cells were cryogenically prepared with the sandwich freeze-fracture technique, as previously described [23], [33], [34]. The SIMS analyses were made in cryogenically prepared fast frozen, freeze-fractured, and freeze-dried cells and pixel-by-pixel subcellular boron isotope image quantification was achieved as described below in the SIMS instrumental parameter section.

### SIMS boron distribution studies of *cis*-ABCPC in F98 rat glioma model

The F98 rat glioma (#CRL-2397, American Type Culture Collection, Manasus, VA) has been used in a wide variety of studies in experimental neuro-oncology [35]. Its biological behavior and infiltrative pattern of growth resemble that of human high grade gliomas. Male Fisher 344 rats weighing ∼200 g were used for *in vitro* uptake studies. F98 glioma cells (10^4^) were implanted stereotactically into the right caudate nucleus of syngeneic Fisher rats. Compound administration and tissue sampling were performed ∼12 days following implantation using three rats per treatment group. The *cis*-ABCPC mixture of L- and D- enantiomers containing ∼ 80% ^11^B and 20% ^10^B (natural abundance) was administered at a dose of 250 mg/kg b.w. via an i.p. injection. In the net boron equivalent, this dose corresponded to ∼16 mg boron from *cis*-ABCPC. The rats were anesthetized using isoflurane and euthanized 2.5 hr. post administration. The brains were removed and the tumors and surrounding brain tissue were frozen in the vapor phase of liquid nitrogen and cryosectioned for correlative histological and SIMS boron distribution analysis.

### SIMS imaging analysis and quantification of subcellular boron distribution

A CAMECA IMS-3f SIMS ion microscope (CAMECA, France) capable of producing isotopic images with a lateral resolution of 500 nm was used in the study [23]. Over the course of its use, this instrument has been extensively upgraded and is equipped with a primary beam mass filter and a 5f Hall Probe control chassis. The control system of the instrument also has been upgraded with a Charles Evans and Associates model PC-1CS Computer Interface system and Windows-based computer. For this study, the instrument was operated in the positive secondary ion imaging mode with samples biased to +4500 V. An O_2_^+^ primary ion beam, accelerated to 10 keV, was focused and adjusted to a nominal beam current of 150–200 nA with a diameter of ∼60 µm when viewed as a stationary spot at the surface of the sample. The primary beam was raster scanned over a 250-µm×250-µm square regions during the analysis. The rate of erosion of tissue specimens was ∼25 Å/s. The 150-µm transfer optics in conjunction with a 60-µm contrast aperture was employed for the cell culture analysis. For tissue imaging, 400-µm transfer optics was used. The energy window of the mass spectrometer was centered and set to a maximal value of 130 eV. Energy and mass filtered secondary ion images were magnified and projected on a single microchannel plate/phosphor screen detection assembly. The gain of the microchannel plate was set at 60% of the maximum. SIMS ion images were recorded from the image detection assembly using a Photometrics CCD CoolSNAP HQ^2^ FireWire Digital Camera capable of 14 bits/pixel image digitization. The image data is transferred from the camera controller to the PC workstation with a Nikon NIS-Elements Imaging Software for storage and digital image processing (Princeton Digital Corp., USA). The camera was operated in the 2×2 binning mode. In the positive secondary ion detection mode, images of isotopes with masses 11, 12, 23, 39, and 40 revealed the subcellular distribution of positive secondary ions of ^11^B, ^12^C, ^23^Na, ^39^K, and ^40^Ca, respectively. High mass resolution analyses confirmed that mass interferences originating from polyatomic ions and cell matrix components were negligible, which is in agreement with our previous studies of BNCT drugs by dynamic SIMS [29], [34], [36], [37]. For the imaging of S-phase cells, the SIMS instrument was first operated in the negative secondary detection mode for imaging of ^81^Br^−^ signals and then the mode of detection was changed to the positive secondaries in the same field of analysis for recording ^39^K, ^23^Na, and ^40^Ca images.

The pixel-by-pixel image quantification of ^11^B^+^ signals was achieved by using ^12^C^+^ carbon normalization approach and the relative-sensitivity-factors (RSF) of boron isotopes to the ^12^C^+^ cell (tissue) matrix signals [23], [36], [37]. This approach included a thorough evaluation of SIMS matrix effects (mass interferences, sputter rate variations, and practical ion yield variations), which were found to be negligible between the nucleus and the cytoplasm of fractured freeze-dried cells [36], [38]. The absolute boron concentrations produced from the SIMS images were converted into estimated wet weight concentrations by assuming 85% water content in cells. The naturally present boron levels in control B16 cells or tumor tissues (i.e. not treated with *cis*-ABCPC) were found to be below the detection limits of SIMS.

### Statistical analysis

A Minitab Statistical Software was used for statistical analysis of the data using ANOVA or the student *t* test. A *p* value of less than 0.05 was considered significant.

## Results

### SIMS imaging of boron distribution from *cis*-ABCPC in the B16 mouse melanoma

**Figure 2:**
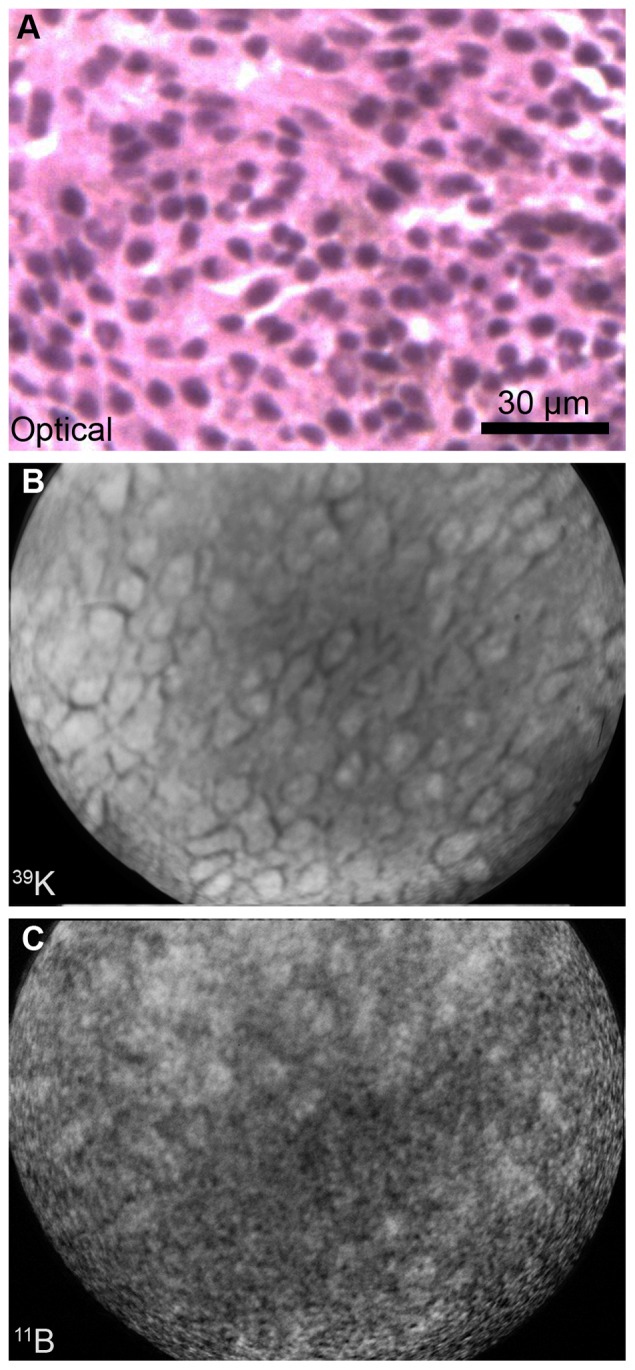
SIMS imaging analysis of ^39^K and ^11^B distributions in B16 melanoma. The B16 melanoma bearing mouse was euthanized 1 hr after i.p. administration of *cis*-ABCPC. The photomicrograph is an H&E stained 4µ cryosection of the B16 melanoma. SIMS images were recorded from the adjacent 4µm thick cryosections. The SIMS ^39^K^+^ image was integrated on the CCD camera for 0.2 sec and ^11^B^+^ image for 2 min.

The B16 tumor morphology is shown in an optical image from a cryosection stained with H & E (Fig. 2A). The tumor is composed of a monomorphic population of cells with hyperchromatic nuclei and cytoplasmic melanin. SIMS imaging analysis from an adjacent cryosection revealed the subcellular distributions of ^39^K^+^ and ^11^B^+^ in B16 tumor tissue (Fig. 2B and 2C). The nuclei of individual cells are discernible in the potassium image (Fig. 2B). In the corresponding boron image, the boron signals are present throughout the cells with a certain degree of heterogeneity between the cells. The nucleus of some cells contained elevated levels of boron (Fig. 2C). Intracellular boron concentrations, obtained from SIMS imaging analysis in all three treatments of 1, 2.5, and 4 hr. post injection of *cis*-ABCPC, are in Table 1. At 1 hr. following post-injection treatment, the tumor cells contained significantly higher (p<0.05) levels of boron, approximately double the boron concentration, than 2.5 and 4 hr. post-injection treatments (Table 1). This is indicative of blood clearance of the compound in mice at longer post-injection time points, and possibly a reduction in the free pool of the trapped *cis*-ABCPC in tumor cells. Since there was no significant difference in boron concentrations in tumor cells between the 2.5 and 4 hr. post-injection treatments, the intracellular boron pool in these time points most likely represented the bound form of *cis*-ABCPC in the cell matrix components of tumor cells.

**Table 1:** Quantitative SIMS imaging analysis of boron concentrations in tumor cells from B16 melanoma bearing mice.

Treatment time (hr)^a^	Boron conc. (µg/g wet weight)	SIMS imaging fields^b^
1.0	28±7^c^	5
2.5	14±5^d^	7
4.0	17±6^d^	7

a Four mice in each treatment received 24 mg boron/kg b.w. *cis*-ABCPC *via* injection. After 1, 2.5, and 4 hr. post injection, the mice were euthanized and samples of tumor i.p. were frozen for SIMS studies. The boron concentrations from SIMS images, as shown in Fig. 2, are expressed in µg/g wet wt. (mean ± SD).

b In the 1 hr treatment group, observations represent 31 regions of interest (ROIs) in 5 SIMS imaging fields. A region of interest within a SIMS imaging field is defined as a clump of 10-15 individual cells taken together for quantification of SIMS images and, therefore, representing a large sampling of the imaging data. In 2.5 hr treatment, observations represent 47 ROIs in 7 SIMS imaging fields. In 4 hr treatment, observations represent 51 ROIs in 7 SIMS imaging fields. The superscript “c” denotes the significant difference (P<0.05) in boron concentrations from treatments designated with superscript “d”.

### SIMS imaging of boron distribution from *cis*-ABCPC in B16 melanoma cells in culture

**Figure 3:**
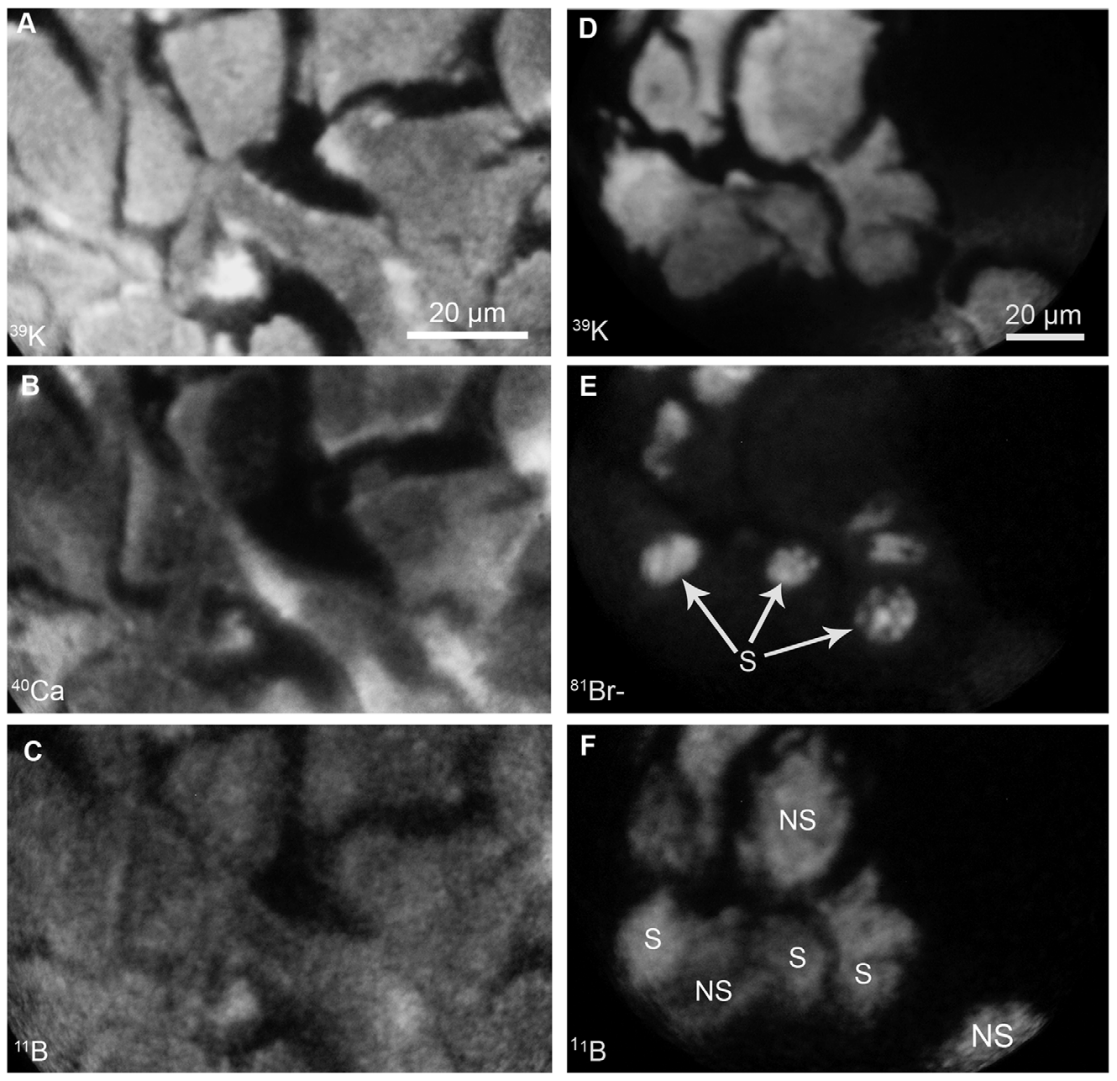
SIMS imaging of boron in B16 cells exposed *in vitro* to *cis*-ABCPC. The cells were treated with 30 ppm boron equivalent of *cis*-ABCPC for 2.5 hr (A-C) and for 1 hr in presence of 50 µM bromodeoxyuridine (BrdU) for the detection of DNA-synthesizing S-phase cells among the asynchronous population by imaging of ^81^Br^−^ signals with SIMS (D-F). The positive secondary ion SIMS images of ^39^K, ^40^Ca, and ^11^B represent the subcellular distributions of potassium, calcium, and boron, respectively, in individual B16 cells (A-C). The ^23^Na image also was recorded (not shown) and it revealed the K/Na ratio of ∼10∶1 in these cells. D-F represent SIMS analyses in *cis*-ABCPC and BrdU treatment. For the imaging of S-phase cells, the SIMS instrument was first operated in the negative secondary detection mode for imaging of ^81^Br^−^ signals and then the mode of detection was changed to the positive secondaries in the same field of analysis. The ^39^K images were integrated on the CCD camera for 0.2 sec. each. The ^40^Ca, ^11^B, and ^81^Br^−^ images were integrated for 2 min. each.

The B16 melanoma cells in culture provided an ideal model for making corroborative observations to the B16 mouse tumor model on the boron uptake and retention from *cis*-ABCPC based on individual cell analysis of the nucleus and the cytoplasm (Fig. 3, panels A-C). Furthermore, the addition of BrdU along with *cis*-ABCPC in the nutrient medium allowed SIMS imaging of ^81^Br^−^ and ^11^B^+^ signals for a direct evaluation of the boron-delivery to the S-phase and the non-S phase cells in the same field of view (Fig. 3, panels E-F). In Fig 3, panels A-C show the distribution of ^39^K, ^40^Ca and ^11^B, respectively, from *cis*-ABCPC treated cells. The individual B16 cells are discernible in the K image, since they are separated by dark intercellular spaces. In the ^40^Ca image the rounded nucleus in each cell was dimly visible, since most of the calcium is stored in endoplasmic reticulum in the cytoplasm. The ^11^B distribution reveals boron from *cis*-ABCPC to be distributed throughout the B16 cells with some degree of heterogeneity. The SIMS analysis of ^39^K, ^81^Br, and ^11^B in BrdU and *cis*-ABCPC treated cells is shown in panels D-F in Fig 3. The S-phase cells (S) among the asynchronous population are identified by incorporation of bromine in their nuclei, as shown in panel E of Fig 3. The boron distribution in the S- and the non-S phase (NS) cells revealed no significant differences (panel F).

Quantitative observations of boron concentrations in the nucleus and cytoplasm of individual cells obtained from SIMS imaging analysis of all treatments of *cis*-ABCPC are summarized in Table 2. The following conclusions can be drawn from these data on uptake characteristics of B16 cells in culture: (i) boron was localized in both nuclei and cytoplasm of the cells with minor differences, (ii) boron uptake in cells increased significantly with longer exposures, as evidenced by partitioning of boron in cell nuclei *vs.* nutrient medium, which had increased from 2.8 at 1 hr. to 7.5 in the 4 hr. treatment samples, and (iii) ∼ 45–50% of the boron pool in both nuclei and cytoplasm was retained in either free or a bound form after a 4 hr exposure, followed by exposure of the cells to compound-free media for 30 min. Conversely, it also was shown that approximately half of the *cis*-ABCPC pool was present in the free form in B16 cells after a 4 hr. exposure to the compound.

**Table 2:** Boron concentration in wet weight in nuclei and cytoplasm of B16 melanoma cells *in vitro* determined by SIMS imaging analysis.

Treatment – 30 ppm Boron equivalent of *cis*-ABCPC^a^	Boron concentration in Nucleus (µg/g mean ± SD)	Boron concentration in Cytoplasm (µg/g mean ± SD)	Nucleus to Nutrient Medium Boron Ratio
1.0 hr	83±18	94±23	2.8
2.5 hr	149±43	162±39	5.0
4.0 hr	225±29	230±50	7.5
4.0 hr followed by 30 min. in Nutrient Medium	102±36	114±30	

a In each treatment, more than 30 cells were analyzed in 5–7 SIMS imaging fields for quantitative analysis.

### SIMS imaging of boron distribution from *cis*-ABCPC in F98 rat glioma

**Figure 4:**
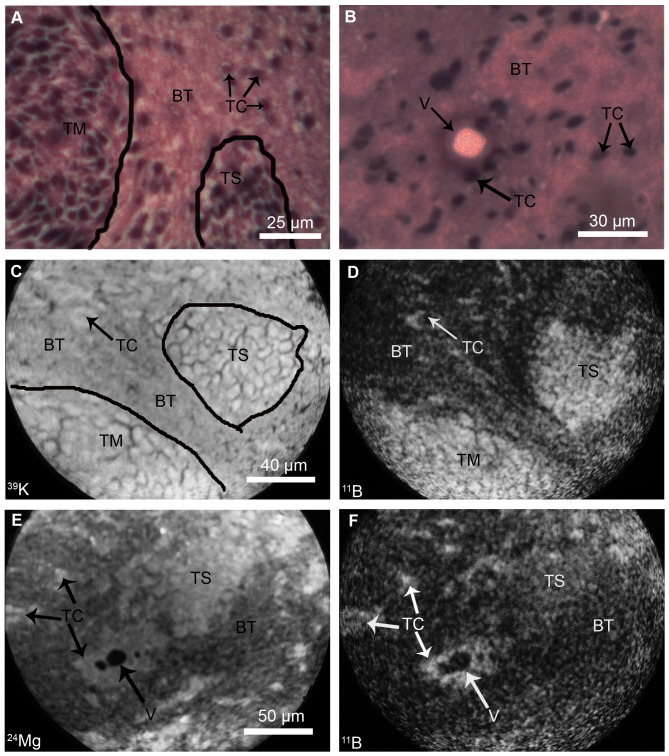
SIMS analyses of brain from F98 glioma bearing rats. Rats received *cis*-ABCPC by i.p. at a dose of 250 mg/kg b.w. and were euthanized 2.5 hr. later. The morphology of F98 glioma is shown in H & E stained cryosections (A-B). The main tumor mass (TM), normal brain tissue (BT), clusters of tumor satellite cells (TS) and individual infiltrating tumor cells (TC) are discernible in normal brain tissue (A). The optical image (B) shows a vessel (V) which is surrounded by TC and BT. SIMS analyses were made in adjacent cryosections. SIMS imaging analyses of boron from two different tissue regions are shown in C-D and E-F, respectively. In C-D, a subtle gradient of ^39^K^+^ in the SIMS image identified the boundary of the TM from the BT which contains a TS and TC’s in panel C. The respective boron microdistribution from *cis*-ABCPC is shown in the ^11^B^+^ boron SIMS image in panel D: the higher concentrations of boron in TM, TS, and TC’s are revealed in comparison to the BT. In a second example of the SIMS imaging of another tissue region (E-F), a gradient of ^24^Mg^+^ is used for the identification of TS and infiltrating TC’s in the normal brain tissue which also contains a vessel (V) surrounded by TC’s (E). The respective ^11^B^+^ SIMS image from this region, shown in F, revealed higher concentrations of boron in TS as well as the infiltrating TC’s in the normal brain tissue surrounding the vessel (V). The image integration times on the CCD camera for SIMS images of ^39^K^+^ and ^11^B^+^ in C and D were 0.2 sec and 2 min, respectively. The image integration times on the CCD camera for SIMS images of ^24^Mg^+^ and ^11^B^+^ in E and F were 1 min and 2 min, respectively.

The F98 rat glioma provided an ideal model for SIMS imaging studies of boron-delivery by *cis*-ABCPC to the main tumor mass, normal brain tissue, and tumor cells infiltrating the normal brain, which are protected by the blood-brain-barrier (BBB). In the F98 rat glioma model, tumor cells may infiltrate normal brain as individual cells or as clusters of cells together, referred to here as the tumor “satellites” [35]. Fig 4 shows morphological evaluation of F98 glioma and SIMS imaging analysis of boron distribution at the level of single cell resolution. In Fig 4, panels A and B show typical histological features of F98 rat glioma observed in optical images of H&E stained cryosections. The main tumor mass (TM), an infiltrating tumor satellite (TS) and individual tumor cells (TC) in normal brain tissue (BT) are discernible. A vessel (V) surrounded by tumor cells is discernible in normal brain tissue (Fig. 4B). Examples of SIMS imaging analyses from adjacent cryosections are shown from two different tissue regions in panels C-F in Fig 4. The panels C and D of Fig 4 show the SIMS analysis of the first tissue region. A subtle gradient of ^39^K^+^ in the SIMS image provided a marker for the boundary of the main tumor mass (TM) from the normal brain tissue (BT) which contains a tumor satellite (TS) and infiltrating tumor cells (TC) (Fig. 4, Panel C). The respective ^11^B^+^ SIMS images from this tissue region revealed higher concentrations of boron in the main tumor mass (TM), tumor satellite (TS) and the infiltrating tumor cells (TC) in comparison to normal brain tissue (BT) (Panel D in Fig. 4).

In Fig 4 panels E and F show the SIMS imaging of the second tissue region. Here, a gradient of ^24^Mg^+^ was used, as reported previously [29], for identification of tumor satellite cells (TS) and infiltrating tumor cells (TC) in normal brain tissue (BT). A vessel (V) surrounded by tumor cells is also discernible (Fig. 4, panel E). The respective ^11^B^+^ SIMS image from this tissue region revealed higher concentrations of boron in tumor satellite cells and infiltrating tumor cells, as well as the tumor cells that surrounded the vessel (Fig. 4, panel F). The normal brain consistently revealed very low boron concentrations, as reflected in the lower intensity darker tissue regions. Taken together, these imaging SIMS observations indicate that *cis*-ABCPC was capable of preferentially delivering higher boron concentrations to F98 glioma cells in the main tumor mass, as well as tumor cells infiltrating the normal brain that are protected by the BBB.

Quantitative observations on boron concentrations from SIMS analyses of *cis*-ABCPC treated F98 glioma bearing rats are summarized in Table 3. Boron concentrations were significantly different (p<0.05) between the main tumor mass, infiltrating tumor cells, and the normal brain tissue. Tumor cells in the main tumor mass were ∼ 8 times higher in their boron content than the normal brain tissue (64 ppm *vs.* 8 ppm, Table 3). The boron partitioning between the infiltrating tumor cells, taken together as cells in tumor satellites and individual infiltrating tumor cells in the normal brain, and the normal brain tissue was observed to be ∼ 5 (Table 3). These observations indicate that: (i) *cis*-ABCPC was capable of delivering boron preferentially to tumor cells in the main tumor mass as well as infiltrating tumor cells in the normal brain, and (ii) the boron content of infiltrating tumor cells in the normal brain tissue was significantly less than the tumor cells in the main tumor mass.

**Table 3:** Quantitative SIMS imaging analysis of boron concentrations^a^ from *cis*-ABCPC in F98 rat glioma brain tissue regions.

Compound	TM	TS & TC	BT	TM/BT	TS and TC/BT
Cis-ABCPC	64±11^b^	41±9^c^	8±3^d^	8.0	5.0

a Boron concentrations are expressed in µg/g wet weight (mean ± SD) in the main tumor mass (TM) and infiltrating clusters of tumor satellite cells (TS) and individual tumor cells (TC) in the normal brain tissue (BT) of F98 glioma bearing rats. The rats were treated with *cis*-ABCPC, as a mixture of its L- and D- enantiomers, with boron equivalent of 250 mg/kg b.w. i.p. for 2.5 hr. The observations represent the sampling of brain tissues from 3 rats and SIMS analysis in 6 imaging fields, as shown in Fig. 4.

b,c,d denote boron concentrations significantly (p<0.05) different from each other.

## Discussion

In the present study, we have evaluated the boron-delivery potential of an UNAA, *cis*-ABCPC as a mixture of its L- and D- enantiomers, at single cell and subcellular scales resolution with a sophisticated technique of dynamic SIMS ion microscopy. The subcellular scale characterization of individual tumor cells at the level of boron imaging in the nucleus is a necessary step in evaluating new candidate compounds as possible boron delivery agents for NCT. Since BNCT is a non-invasive therapeutic modality, which has the potential of killing the individual infiltrating tumor cells in normal brain, it is essential that a new compound be rigorously tested for its efficacy in appropriate models to allow such single cell studies. Indeed, the present single cell boron imaging study is an extension of our recent work on *cis*-ABCPC on homogenized tissue measurements with inductively coupled plasma–optical emission spectroscopy (ICP-OES) [22]. In this study we found that: (i) *cis*-ABCPC was comparable to the clinically used compound BPA in delivering the net boron content to B16 and F98 tumors, as measured by means of ICP-OES, and (ii) *cis*-ABCPC was far superior to BPA in providing a higher boron partitioning ratio of tumor-to-blood in B16 mouse melanoma model and the main tumor mass-to-healthy brain tissue boron ratio in the F98 rat glioma model [22]. The later indicates a highly desirable feature of *cis*-ABCPC as a boron delivery agent. The present study has defined single cell delivery of *cis*-ABCPC and has established its ability to target tumor cell nuclei, S-phase of the cell cycle, and infiltrating tumor cells in normal brain that are protected by the BBB.

SIMS studies of B16 murine melanoma revealed that boron from *cis*-ABCPC is distributed throughout the cell, including nuclei, and that there was some degree of *in vivo* heterogeneity among individual tumor cells (Fig 2, Table 1). SIMS observations also demonstrated that following i.p. injection of *cis*-ABCPC to B16 melanoma bearing mice tumor cells attained the highest boron concentrations within an hour and then boron levels fell to ∼50% after 2.5 and 4 hrs. following administration (Table 1). This most probably was due to clearance of the compound from the vascular compartment [22]. These observations suggest that approximately half of the boron pool from *cis*-ABCPC in B16 tumors was present in some form of bound form. This is a positive feature for BNCT, since there should be a sufficient time interval between drug administration and irradiation in order to reduce the blood boron concentration to a safe level [1]. The presence of a significant pool of bound boron in tumor cells may not be significantly reduced under such conditions. Furthermore, our *in vitro* studies with B16 cells revealed that a longer exposure to *cis*-ABCPC resulted in higher boron uptake. Furthermore, the compound was capable of delivering boron to tumor cells in all phases of the cell cycle, including S-phase cells (Fig. 3, Table 2). The boron retention study of B16 cells *in vitro* further confirmed the SIMS observations of tumors from B16 melanoma bearing mice. As the exposure of *cis*-ABCPC-treated cells to the drug-free nutrient medium quickly removed approximately half the boron pool within 30 min. (Table 2). SIMS images from the retention study demonstrated that the remainder of the boron pool was distributed throughout the cell, including nuclei (data not shown). Taken together, these observations suggest that standardization of the drug-delivery protocol (i.e., i.p. injection, i.v. infusion, and optimal time interval for boron uptake) for *cis*-ABCPC could further enhance its potential to selectively deliver boron to tumor cells.

The challenge in treating high grade gliomas by any therapeutic modality is that the tumor is highly infiltrative of normal brain. These infiltrating tumor cells are protected by an intact BBB and spread throughout the brain. Since the infiltrating tumor cells cannot be surgically removed, they provide focal deposits for tumor regrowth. BNCT can selectively kill infiltrating tumor cells in the normal brain, if they contain a sufficient number of ^10^B atoms with high selectivity in comparison to the normal brain tissue. To date, in clinical BNCT of high grade gliomas, the blood boron concentration has been used to calculate the tumor concentration using a “compound factor”. Based on this it has been assumed that the infiltrating tumor cells contain identical ^10^B concentrations as the main tumor mass. Since there are no currently available methods, including MRI and PET scan, with the requisite sensitivity and spatial resolution to determine in real time the tumor boron concentrations, dosimetry for BNCT is based on computational models [1]. Although SIMS cannot be used under clinical conditions due to its high vacuum requirements, it provides an invaluable tool for studying boron concentrations with sufficiently high resolution to detect individual and clusters of infiltrating tumor cells in the normal brain tissue in cryogenically fixed samples.

In this study, SIMS revealed that *cis*-ABCPC delivered higher boron concentrations to F98 glioma cells in the main tumor mass, as well as infiltrating cells and clusters of tumor cells in satellites in comparison to the normal brain tissue (Fig. 4, Table 3). However, the tumor cells in the main tumor mass contained significantly higher boron concentrations (p≤0.05) than infiltrating tumor cells in the normal brain tissue (64±11 ppm boron *vs.* the 41±9 ppm boron, mean ± SD, Table 3). Such a difference indicates that the BBB played a significant role in restricting drug delivery to infiltrating tumor cells in the normal brain tissue, which are protected by an intact BBB. Although the main tumor mass of gliomas may have a leaky BBB [39], as reported by Barth and his research team, disruption of the BBB by the administration of a hyperosmotic solution of mannitol, significantly increased the uptake of BPA in F98 glioma bearing rats [40], [41]. As recently described, the use of focused ultrasound to disrupt the BBB resulted in enhanced tumor uptake of BPA in 9L gliosarcoma bearing rats [42]. SIMS observations in the present study on *cis*-ABCPC are also consistent with previous studies on BPA that demonstrated lower boron concentrations in infiltrating F98 glioma cells dispersed in normal brain compared to tumor cells in the main tumor mass in the F98 and other rat glioma models [26], [29], [37], [42], [43]. The exact mechanism by which *cis*-ABCPC crosses the BBB is not known, but these types of unnatural cyclic amino acids have been shown to cross the intact BBB due to the presence of L-amino acid transferase [44]. A better partitioning of boron from *cis*-ABCPC might reflect its lower uptake in normal brain tissue and higher uptake in infiltrating tumor cells. Since L-type amino acid transporters (LAT) are up-regulated in high grade gliomas [45], [46], the L isomer of *cis*-ABCPC is likely to accumulate in higher concentrations in infiltrating tumor cells compared to surrounding normal brain tissue, as shown in SIMS images (Fig. 4, Table 3).

*Cis*-ABCPC is far superior to BPA because it provided higher boron-partitioning between infiltrating tumor cells and normal brain. A boron-partitioning ratio of 5:1 was observed for *cis*-ABCPC (Table 3) compared to 1.5–2.0∶1 reported for BPA in the 9L rat gliosarcoma [37], F98 glioma [29], [43], and C6 glioma models [26]. The *cis*-ABCPC has a high selectivity to infiltrating tumor cells, as reflected in 5:1 boron ratio between the infiltrating tumor cells and the normal brain tissue (Table 3). This is a novel feature of *cis*-ABCPC for BNCT of high grade gliomas, since radiation damage to normal brain tissue can be significantly reduced if the quantity of boron in infiltrating tumor cells exceeds that found in surrounding normal brain by at least a factor of three [6], [7]. Once trapped inside the tumor cells, the *cis*-ABCPC is water soluble and cannot be metabolized, which may further enhance cellular retention compared to BPA, which is metabolized [47]. In conclusion, the *cis*-ABCPC, as a mixture of L- and D-enantiomers, potentially would be a novel boron delivery agent for melanomas and high grade gliomas. The subcellular SIMS imaging observations, discussed in the present study, provide compelling support for further evaluation of this compound to optimize its dosing paradigm and define its toxicity in large animal models of high grade gliomas and melanoma metastatic to the brain. Finally, it should be noted that any therapy studies will require the synthesis of ^10^B enriched *cis*-ABCPC.
